# The Surgical Problem Presented by the Acutely Distended Abdomen

**Published:** 1910-09-17

**Authors:** Ivor Back

**Affiliations:** Assistant Surgeon to St. George's Hospital.


### Hospital Clinics.

### THE SURGICAL PROBLEM PRESENTED BY THE ACUTELY DISTENDED ABDOMEN.

By IVOR BACK, M.A., M.B., B.C.Cantab., F.R.C.S.Eng., Assistant Surgeon to St. George's Hospital.

(A Clinical Lecture delivered at St. George's Hospital.)

I have chosen the surgical aspect of the acutely distended abdomen as the subject of my clinical lecture this afternoon for several reasons. You do not often see these cases when you are students, because they are sent up to the hospital at irregular hours as emergencies, and you have, therefore, few opportunities of investigating them or of inquiring into their history: you are compelled to rely on text-books of surgery for your knowledge of the condition. Now in most text-books this subject is treated from an academical, rather than from a practical, point of view; so that, although you may know all the causes which give rise to acute abdominal distension you are rarely left with a clear idea of the measures which you should take if you were suddenly called upon to deal with such a case.

I need hardly remind you that the causes of abdominal distension are manifold: it may result from a collection of fluid, as in cirrhosis of the liver, or from the presence of an ovarian cyst, or of a hydronephrotic kidney. You will find an account of such a case in Bland-Sutton's "Tumours." The patient was a girl named "Mary Nix, who died at Hampton-Poyle, near Oxford, at the age of twenty-three. She had a hydronephrosis containing fluid to the amount of thirty gallons, wine measure. The dissection of the body was conducted by Samuel Glass, with some learned gentlemen of the University." But I think it may be said without contradiction that acute abdominal distension is most often the result of an obstruction to the passage of some portion of the alimentary canal: and it is of distension arising from this cause that I am about to speak.

We are accustomed to divide intestinal obstruction into two forms—(*a*) acute and (*b*) chronic. The more acute the process the less likelihood there is of an extreme or even of a noticeable degree of abdominal distension. You must have noticed that in a case of strangulated hernia you have rarely seen the abdomen distended. The explanation lies in the very acuteness of the trouble; for the patient either succumbs or is relieved by operation before the abdomen has had time to become distended. But there is an exception to this rule. Internal strangulation of the alimentary canal—and by strangulation I refer to an acute obstructive process which not only occludes the lumen of the gut but which also damages the vessels which supply it—is sometimes associated with a rapid onset of distension, which is due in this case to the accumulation of gases in the affected coil; the damage to the vascular supply interferes with the power of absorption, which is one of the normal functions of the capillary vessels. Rapid distension of this nature is known as "meteorism." It is particularly associated with a volvulus of the pelvic colon. The illness starts with the sudden onset of acute abdominal pain, accompanied by vomiting. This initial vomiting is due not to any mechanical obstruction, but to shock following interference with the splanchnic ganglia. The abdomen is at first quite flaccid, the pulse small and slow, and the temperature sub-normal. Later—and this may be a matter of a few hours—abdominal distension occurs, localised in this case to the left lower quadrant of the abdomen. Vomiting recurs and becomes persistent, at first gastric, then bilious, and finally stercoraceous. The pulse becomes quick and irregular, but the temperature remains subnormal. Such a syndrome will render the diagnosis comparatively easy. But you will have far more difficulty in those cases in which the abdomen has become progressively distended as the result of a chronic intestinal obstruction which is gradually becoming absolute.

The increased proportions of the abdomen depend partly upon an accumulation of intestinal contents and partly upon meteorism. The degree of distension varies with the site and narrowness of the stenosed area. When meteorism is the more marked feature the abdomen is universally prominent and tympanitic; but when the distension is due to intestinal contents alone the loaded coils of intestine tend to gravitate into the loins when the patient is recumbent, and the wide abdomen so produced is not unlike that of ascites.

Two distinct problems will confront you: (1) In what part of the alimentary canal is the stenosis situated; and (2) what is the character of the lesion causing the stenosis?

In certain favourable cases a definite diagnosis may be made, but in the great majority a positive opinion cannot be given. Surgical diagnosis is based upon accumulated experience, and one observes that the diffidence of a surgeon is to a certain extent directly proportional to the amount of his clinical knowledge. Hard and fast rules cannot be laid down in surgery, because individual cases tend to exhibit unsuspected and surprising features.

I have said, however, that a definite diagnosis may be made in certain cases. They are those in which a digital examination of the rectum reveals not only the situation but the exact nature of the obstruction. It is a regrettable fact, but none the less a true one, that this method of examination is often omitted. Too much stress cannot be laid upon its importance, not only in cases where the symptoms are directly referable to the rectum, but whenever they point to a pelvic origin. Many obvious errors of diagnosis will be avoided if this precaution is taken. I might mention in passing only a few of the conditions in which information may be obtained in this way, although the rectum itself is not diseased. The diagnosis of an appendix abscess may be made certain if a hot tender swelling is found pressing upon the right wall of the rectum. Similarly much information may be obtained as to the condition of the vesiculæ seminales and prostate, as might be expected from their intimate relation to the anterior rectal wall; in fact, by this means alone can it be determined if they are involved in a tuberculous infection which began in the epididymis. And last, but not least, whenever tuberculous disease affecting the lower part of the vertebral column is suspected, a rectal examination should be made. The symptoms in these cases are often vague, while all the time there may be a large abscess filling the pelvis and pressing on the rectum.

A positive diagnosis may also be made by means of the sigmoidoscope, when the obstruction is situated in the lower part of the sigmoid or in the first part of the rectum. But, apart from these isolated instances where the condition may be diagnosed with more or less certainty, it is generally impossible to do more than to attempt to differentiate stenosis of the small intestine from that of the large.

To do this a thorough examination of the abdomen is most important. In general the position and arrangement of the distended portions of intestine correspond to their normal position. It is, therefore, more important to study their arrangement than their size or circumference; and it is a good rule in attempting to identify a particular loop of intestine through the parietes to ascribe it to that part of the intestinal canal which would normally occupy the position of the loop. The coils most easily identified in this way are the transverse colon and the sigmoid. Obstruction in the sigmoid often causes a distension of the lateral aspects of abdomen, and in these cases percussion in the lumbar region will give a resonant note on either side of the vertebral column. The resonance may be unilateral from one of two causes: it may be confined to the left side in sigmoid obstruction when as yet only the descending colon is dilated, or to the right side when the transverse colon is the seat of the obstruction.

In small intestine obstruction on the other hand, there is no bulging of the flanks, and the distended coils form a rounded protuberance in the centre of the abdomen. The value of this information was demonstrated in this hospital recently. A case was admitted with symptoms of intestinal obstruction under the care of one of my colleagues. A rectal examination was made and a carcinomatous stricture discovered; this was still pervious though the lumen was very small. The abdomen was distended in the centre but not in the flanks. The surgeon was therefore of opinion that the rectal condition did not account for the symptoms entirely, but that there was also some obstruction in the small intestine. Laparotomy was performed, and a gall-stone was found impacted in the lower end of the ileum.

As a contrast to this case I will mention one in which I was misled by an imperfect appreciation of the physical signs which I found on examination of the abdomen. The patient was a French girl, aged 25, who was admitted to this hospital last year. Three days before admission she had been seized with diarrhœa, and the next day she had an acute pain all down the left side of the abdomen, which had persisted. Since the onset she had vomited everything she had eaten. There was no history of previous indigestion. On admission she locked ill, and complained of severe pain in the left half of the abdomen. She had an anxious look, and her eyes were sunken. Her pulse was 160, feeble, and her temperature 96°. The upper three-fourths of the abdomen were much distended and appeared to be occupied by an enormous ovoid mass, whose long axis was vertical. The upper part of the abdomen did not move on respiration, and there was distinct resistance on palpation. On percussion not only was the whole mass itself resonant, but the normal liver dulness was completely absent, except ior a small area in the right axillary line. A vaginal examination was made, but it revealed no abnormality of the pelvic viscera.

I came to the conclusion that the condition was one of peritonitis following a perforated gastric ulcer, and that the mass was probably composed of paralysed and distended coils of small intestine. I opened the abdomen by a vertical incision in the left epigastrium and found that the swelling felt per abdomen was an abnormally dilated stomach. The upper pole had pushed the liver out of reach under the costal arch, while the lower was resting on the fundus uteri. I opened the swelling and evacuated seven pints of greenish yellow fluid. The wound in the stomach was sewn up with Lembert sutures. The patient made an uneventful recovery. It is proverbially easy to be wise after the event, but I think now that I ought to have suspected the true state of affairs on account of the localised character of the swelling and the history of incessant vomiting without hæmatemesis. Had I arrived at a correct diagnosis I could, of course, have effected a cure by passing a stomach tube.

You know that in chronic obstruction the peristaltic movements of the hypertrophied intestine are often visible through the abdominal parietes. The character of these may aid you in the diagnosis of the site of the obstruction. They occasionally lead up to one part of the abdomen, such as the ileo-cæcal valve, with definite regularity and stop there. When this occurs it may be concluded that the obstruction is in the neighbourhood of the cæcum, especially if the movements resemble small intestine movements—that is, if they are quick and restless. But it must be remembered that if the intestine above the obstruction is tired and paralysed from over-work and over-distension, the movements may stop at a point which is situated at some distance from the actual obstruction.

A careful study of the symptoms of which the patient complains may give valuable information. Pain, however, is not of much diagnostic significance in the exact localisation of the obstruction, since it is more often due to colic than to the actual obstruction and is sometimes referred to remote parts of the abdomen in a most misleading way. The symptoms of obstruction of the small intestine are said to come on earlier and to be more violent and more persistent.

Vomiting also appears earlier in stenosis of the small intestine and is more distressing and more constant. It tends to become stercoraceous on about the fifth day after the obstruction has become absolute. In obstruction of the colon it is a late feature, is scanty, and is usually not stercoraceous until shortly before the death of the patient.

Tenesmus and the passage of blood in the stools are highly characteristic of disease in the colon, while the presence of indican in the urine is more suggestive of obstruction in the small intestine.

The recognition of the nature of the lesion in a case of abdominal distension due to intestinal obstruction is important, because the prognosis you will give must to a large extent depend on this, and it will influence you in determining the exact procedure that you will adopt. Success in making the diagnosis depends upon the thoroughness of your examination. Let me first impress upon you the importance of examining all the hernial orifices in every case of persistent vomiting. It is unsafe to assume that the patient will tell you that he has a hernia. He may not know it himself; or, even if he does, it is possible that he will not mention it, because it does not occur to him that his present symptoms can be connected with it. I can recall a case which was treated as one of gastritis for more than twenty-four hours before it was discovered that the *fons et origo mali* was a small obstructed femoral hernia. In women it is equally important to make a vaginal examination. Enlargement or displacement of some portion of the genital apparatus may compress the rectum against the unyielding walls of the long pelvic canal.

The age of the patient should be considered and he should be examined for the signs of the cachexia of malignant disease, especially if he is over the age of forty. No one symptom is pathognomonic, but the combined onset of anæmia, emaciation, and gradual loss of weight are profoundly suggestive of carcinoma. Personally, I attach much importance, as a diagnostic feature, to a loss of the natural elasticity of the skin, so that if it be pinched up at any point there is an appreciable interval of time before it returns to its normal condition.

It is not my purpose to-day to describe in detail the various operative procedures which are at your disposal for the relief of intestinal obstruction, nor shall I discuss the treatment of those cases in which the diagnosis can be made certain, as in carcinoma of the rectum. I desire merely to call your attention to the consideration of certain aspects of the problem which confront the surgeon when he is about to perform laparotomy for the relief of an intestinal obstruction, the situation and nature of which are both uncertain.

The first question which naturally presents itself is, " What incision shall be made? " I am strongly of opinion that the most useful incision is a vertical one at the outer edge of the right rectus abdominis muscle, with its centre situated below the umbilicus. As soon as the peritoneal cavity is opened you can explore the condition of the cæcum, and you are immediately in possession of invaluable information. If the cæcum is collapsed, it is obvious that the site of the obstruction is on its proximal side—*i.e.* in the small intestine—whereas if it is distended it may be taken for granted that some part of the colon is primarily at fault. This will enable you to direct the subsequent steps of your exploration.

Now you are told in text-books of surgery to search for the obstruction with a hand in the wound without letting the distended coils emerge from the abdomen. I doubt if you will often succeed in doing this, however great your dexterity, if for no other reason than this, that the distended intestines have a way of extruding themselves without your permission and in spite of your efforts to retain them in position. I would advise you to allow them to come out quite freely. No possible harm can be done to them provided that you allow them to prolapse into sterilised towels, wrung out in hot saline solution. You can then search for the lesion in comparative comfort, for when the small intestine is out of the way the whole of the colon can be adequately exposed, while if the trouble is in the small intestine itself you can pass it through your hands and examine it *seriatim*.

There is a further advantage in making your incision in the right semilunar line. Apart from the rectum and sigmoid, which are both common seats of carcinoma, the most frequent situation of an intestinal obstruction is at or near to the cæcum. This will be obvious to you if you consider for a moment all the possible causes of obstruction in this neighbourhood—strangulation of the ileum by an adherent Mechel's diverticulum, or by adhesions resulting from a past attack of appendicitis which has undergone a spontaneous cure, impaction of a foreign body, such as a gall-stone, at the ileo-cæcal valve, or a retroperitoneal hernia in one of the para-cæcal diverticula. I had under my care last year a case of intestinal obstruction following on the spontaneous cure of an appendix abscess. The patient, a man aged fifty-four, sent for me in July 1909. In January he had been seized with sudden acute pain in the right iliac fossa. He was treated by a doctor—who told him that he was suffering from appendicitis—with morphia and the application of turpentine stupes to the abdomen. He was exceedingly ill and for a fortnight his life was despaired of, but eventually he began to recover. Great difficulty was experienced in getting his bowels to act. His convalescence was tedious and he remained in bed until April. The difficulty with the bowels continued and he had occasional attacks of abdominal distension and vomiting. On July 16 he became suddenly worse; the constipation became absolute and he had persistent vomiting, which became stercoraceous. When I saw him he looked very ill. The abdomen was distended in the centre. Peristalsis was not visible. Rectal examination was negative. His temperature was 96° F. and his pulse 120. I opened the abdomen in the right semilunar line. In the neighbourhood of the cæcum the intestines were matted together in a dense mass of adhesions, some of which were a quarter of an inch in diameter. I performed a lateral anastomosis of the ileum to the ascending colon, but the patient died six hours after the operation. The autopsy revealed further extensive adhesions in the pelvis which were binding the sigmoid and rectum to the bladder. It appeared that the patient must have had an enormous appendix abscess, which had been entirely absorbed without surgical interference. It seems incredible, but the ways of appendix abscesses are passing strange. I have come across one case in which an equally large abscess burst into the cæcum and was discharged per rectum. The patient recovered.

The next problem which will confront you is to determine what you will do when you have found the obstruction. In some cases this is easy. If, for instance, the small intestine has been kinked by a peritoneal adhesion, it is your obvious duty to divide this and to free the kinked coil: if the intestine has not been severely damaged at the point of the obstruction the lumen of the alimentary canal is restored and the operation is complete. But, unfortunately, all cases are not as simple as this. Suppose, for example, you find a carcinoma of the splenic flexure of the colon; what are you going to do? Do not attempt to excise the growth at the first operation. Your efforts will be doomed to failure, for you must remember that the operation, which is at all times an exceedingly difficult one, is doubly so now on account of the distension of the intestine, and, further, that a patient who is suffering from absolute obstruction is ill-fitted to stand the shock of such a procedure. Be content to relieve his obstruction in the quickest and simplest possible manner. You can do this by opening the cæcum and tying in a Paul's tube. When the intestinal contents have drained away, you can return the intestines to the abdomen. The cæcum should then be fixed into the lower portion of the wound by sutures which pass through the serous and muscular coats of the bowel and then through the parietal peritoneum. The rest of the wound should be sewn up. If the patient recovers, the advisability of a second operation can be considered subsequently, and you will be able to weigh the respective merits of transverse colostomy, lateral anastomosis of transverse to descending colon, or excision of the growth, because you have obtained at the first operation an exact knowledge of the state of affairs with which you have to deal.

But now let us suppose that you find a mass of adhesions binding down and obstructing the ileum in the region of the cæcum, so dense that to attempt to liberate the gut and restore its lumen is out of the question. You are face to face with the same problem as in the last case: whether to attempt to restore the patency of the alimentary by a lateral anastomosis, which is a severe operation, or whether you should be content merely to relieve the obstruction for the time being. I am sure that the latter is the proper course. I have told you of a case of intestinal obstruction following the spontaneous cure of an appendix abscess, in which I performed a lateral anastomosis. I will confess to you that I now think I was wrong to do this. I should have been content to perform a temporary enterostomy.

The technique of an enterostomy in the small intestine is essentially different from that of a colostomy. The latter is a permanent measure: the colon is therefore brought out of the wound and fixed there by means of a Spencer-Wells forceps passed through its mesentery, so as to form a spur over which the intestinal contents are discharged to the exterior. But an enterostomy is necessarily a temporary proceeding, because, as I need hardly remind you, the presence of an artificial anus in the small intestine is incompatible with the prolongation of life for an indefinite period. The average duration has been estimated at between two and three months. Until comparatively recent times enterostomy was rarely performed for the temporary relief of obstruction, and surgeons had a great prejudice against it. This was due to the fact that the operation was wrongly performed: the small intestine was brought out and fixed in the wound in the same way as in a colcstomy. It was then found that it was exceedingly difficult to close the opening subsequently. You will not experience this difficulty if you do an enterostomy in the way I advise. I take a piece of rubber tubing and evert the lower half-inch so that it is turned back on itself in the form of a cuff. This makes an admirable Paul's tube, and it has the advantage of being less irritating to the intestine than glass, besides being easier to tie in firmly. An incision is made in the distended intestine just large enough to admit the tube, the end of which is inserted to the limit of the cuff. It is then tied in with two purse-string sutures, the first going through all the layers of the gut and the second only through the serous and muscular. The contents of the distended intestine are allowed to drain away, and the intestine in the neighbourhood of the tube is fixed to the parietal peritoneum with stay sutures, exactly as in a cæcostomy. The rest of the wound is sewn up. In a successful case the enterostomy is shut off by adhesions in a few hours, and the tube works its way out in a week.

In another week the second operation for the permanent cure of the obstruction may be undertaken. The wound is re-opened and the adhesions which attach the enterostomy to the parietal peritoneum are freed, care being taken that there is no escape of fæcal matter while this is being done. The wound in the intestine is sewn up with Lembert sutures. A lateral anastomosis can then be performed under circumstances infinitely more favourable than in the first instance.

In conclusion, I have merely attempted to point out to you some of the difficulties which will confront you in the diagnosis and treatment of cases of intestinal obstruction in which abdominal distension has supervened before you see them. My chief anxiety has been to convince you that it is your duty in these cases to relieve the acute obstruction at a preliminary operation and to postpone any radical proceeding, if you consider one necessary, to a date when the patient and his intestine are both in as good a condition as possible.

